# Number of Tuberculosis Tests and Diagnoses of Latent Tuberculosis Infection Among U.S. Army Active Component Service Members, January 2014–December 2023

**Published:** 2026-03-06

**Authors:** Ralph A. Stidham, Rachel G. Tyler

**Affiliations:** Epidemiology and Disease Surveillance, U.S. Army Public Health Command, West, Joint Base San Antonio–Fort Sam Houston: Dr. Stidham; Army Public Health Nursing, U.S. Army Public Health Command, West: LTC(P) Tyler

## Abstract

Tuberculosis (TB) remains a force health protection threat to the U.S. military, particularly in crucial populations at increased risk of exposure or re-activation. This analysis examined TB testing trends and the prevalence of latent tuberculosis infection (LTBI) among U.S. Army active component soldiers from 2014 through 2023, the first decade following a major policy shift to targeted testing. Defense Medical Surveillance System data indicate that a total of 339,465 TB tests were administered, primarily (81.0%) tuberculin skin tests. Of those tests, 22,762 (6.7%) were positive, leading to the identification of 18,018 (5.3%) LTBI diagnoses. Asian / Pacific Islander soldiers demonstrated the highest LTBI diagnosis proportion (10.2%), followed by non-Hispanic Black (8.6%), Hispanic (5.6%), and Non-Hispanic White (2.9%) soldiers; the data also include ‘other’ (6.8%) and ‘unknown / missing’ (3.6%) categories. Recruits exhibited a significantly higher LTBI diagnosis proportion (11.0%) than non-recruits (3.6%), highlighting a high prevalence of LTBI among incoming personnel at time of accession. A marked decline in testing volume—a 72% decrease from 2014 to 2023 in the annual numbers of tests administered—followed the 2013 U.S. Army Medical Command policy shift. The substantially higher average proportion (6.7%) of positive tests from 2014 to 2023 compared to the average from the pre-policy era (1.3%) of universal screening demonstrates the successful concentration of testing resources on those most at risk, thereby improving diagnostic yield within a low-prevalence military force. This analysis's findings describe the epidemiological outcomes of the Army's targeted testing policy and under-score the importance of ongoing, targeted surveillance to mitigate TB risks in military settings.

Tuberculosis (TB) remains a significant force health protection concern for the U.S. military, primarily due to the risk of activating latent tuberculosis infection (LTBI) and the potential for transmission in congregate settings. ^1 , 2^ A 2014 analysis in *MSMR* of TB testing in all branches of the U.S. Armed Forces, covering the period from 2004 through 2012, provides a critical baseline for the present analysis. During that era of routine annual screening, the prevalence of LTBI diagnoses was low and stable, ranging from just 0.9% to 1.6% annually among those tested. ^3^ That report provides the context for the current analysis, which focuses on the U.S. Army in the decade following a major policy change.

**What are the new findings?**

The 2013 policy that successfully transitioned the U.S. Army from universal tuberculosis screening to a targeted, risk-based strategy reduced testing volume by 72% over the next decade. The decline in tuberculosis testing volume coincided with a substantial increase in diagnostic yield, with the overall positivity proportion rising from 1.3% in the pre-policy era to 6.7% in 2023.

**What is the impact on readiness and force health protection?**

The 2013 policy revision to a targeted, risk-based tuberculosis testing strategy succeeded in focusing valuable public health resources on high-risk groups. The high prevalence (14.0%) of latent tuberculosis infection that has been identified in recruits confirms that accession is the most critical juncture for tuberculosis control within the Army. Slight but notable differences in testing type positivity suggests opportunity for further policy refinement.

In November 2013, the U.S. Army Medical Command (MEDCOM) published Regulation 40-64, *The Tuberculosis Surveillance and Control Program* , which fundamentally altered the Army's approach to TB control. ^4^ This directive shifted the strategy from universal annual testing to a targeted, risk-based testing model, aligning with modern public health principles advocated and then formally updated in May 2019 by the U.S. Centers for Disease Control and Prevention (CDC) and National Tuberculosis Controllers Association (NTCA), which revised sections of previous guidelines. The rationale for this change was to improve screening efficiency and reduce the high number of false positive results when testing large, low-prevalence populations, thereby avoiding unnecessary follow-up procedures and resource expenditures. ^4 , 5^

Under the targeted testing policy, routine TB testing is discouraged and is instead mandated only after a formal risk assessment. Key high-risk populations designated for testing include 1) all new recruits upon accession into service, 2) personnel who have deployed or traveled to TB-endemic regions, 3) individuals identified as close contacts of an infectious TB case, and 4) personnel with specific clinical or occupational risk factors, such as health care workers. ^2 , 4^ The objective of this analysis was to describe the trends of TB tests and LTBI positivity in Army active component soldiers from January 2014 through December 2023, the first full decade following the implementation of this targeted testing policy, and to compare these findings to the pre-2013 baseline.

## Methods

The analysis population included all Army active component soldiers who had a TB test at any military hospital or clinic from January 2014 through December 2023. The data source was the Defense Medical Surveillance System (DMSS). Tests for TB were identified using a combination of immunizations, laboratory, and outpatient procedure data. The DMSS includes data for Army active and reserve component soldier immunizations received during military service and administrative (i.e., billing records) from inpatient and outpatient medical encounters for all Military Health System (MHS) beneficiaries when reimbursed through TRICARE. Laboratory data for interferon gamma release assays (IGRAs), which include QuantiFERON-TB Gold Plus (QFT) and T-SPOT. QFT and T-SPOT tests are IGRAs used to detect TB infection; QFT measures overall amount of IFN-γ, or interferon gamma, while T-SPOT counts number of cells producing IFN-γ. TB tests performed during the surveillance period were provided by the Defense Center for Public Health–Portsmouth. All laboratory tests were classified as IGRA. Tuberculin skin tests (TSTs) were identified from immunizations or outpatient procedures, as depicted in **Table 1** . Out-patient procedures were used to identify additional IGRA tests **(Table 1)** . When calculating the number of tests administered, an individual was counted once per day.

**TABLE 1.** Diagnostic Codes for Tuberculosis and Latent Tuberculosis Screening, Testing and Diagnosis, U.S. Army Active Component, 2014–2023

Diagnostic Code	Description	Test Type ^a^
CVX (non-vaccine)		
095	Tuberculin skin test, old tuberculin multipuncture device	TST
096	Tuberculin skin test, purified protein derivative solution, intradermal	TST
097	Tuberculin skin test, purified protein derivative solution, multipuncture	TST
098	Turberculin skin test, NOS	TST
Outpatient CPT		
86580	Skin test; tuberculosis, intradermal	TST
86480	Tuberculosis test, cell mediated immunity measurement of gamma interferon antigen response	IGRA
86481	Tuberculosis test, cell mediated immunity measurement; enumeration of gammainterferon-producing T-cells in cell suspension	IGRA
86585	Skin test; tuberculosis, time test	TST
ICD-9-CM		
795.5, 795.51, 795.52	Latent tuberculosis	
010 ^*^ –018 ^*^	Active tuberculosis	
ICD-10-CM		
22.7, R76.11, R76.12. Z86.15	Latent tuberculosis	
A15 ^*^ –A19 ^*^	Active tuberculosis	

Abbreviations: CVX, vaccine administered; TST, tuberculin skin test; NOS, not otherwise specified; CVT, Current Procedural Terminology; TB, tuberculosis; IGRA, interferon gamma release assay; ICD-9-CM, International Classification of Diseases, 9th Revision, Clinical Modification; ICD-10-CM, International Classification of Diseases, 10th Revision, Clinical Modification.

* Codes from International Classification of Diseases include a range of codes within specified category.

a If CPT 86580 or 86585 or CVX code 095, 096, 097 or 098, then test type TST; if CPT 86480 or 86481 or if record from laboratory data, then test type IGRA.

For the purposes of this analysis, a ‘positive’ test was any TST or IGRA test result recorded as “positive” in the data-base. A diagnosis of LTBI was defined as an individual with a record of a positive TB test who also received a corresponding International Classification of Diseases, 9th or 10th revision, Clinical Modification (ICD-9-CM / ICD-10-CM) code for LTBI (ICD-9-CM: 795.5x; ICD-10-CM: R76.11, Z22.7) **(Table 1)** in any diagnostic position within 30 days of the test. Demographic information was identified at the time of each test, including beneficiary type, age, sex, race or ethnicity, branch of service, and geographic region of the military treatment facility performing the TB test.

Under the post-2013 targeted testing policy evaluated in this analysis, Army personnel were eligible for TB testing based on a risk assessment.

## Results

**FIGURE 1.**
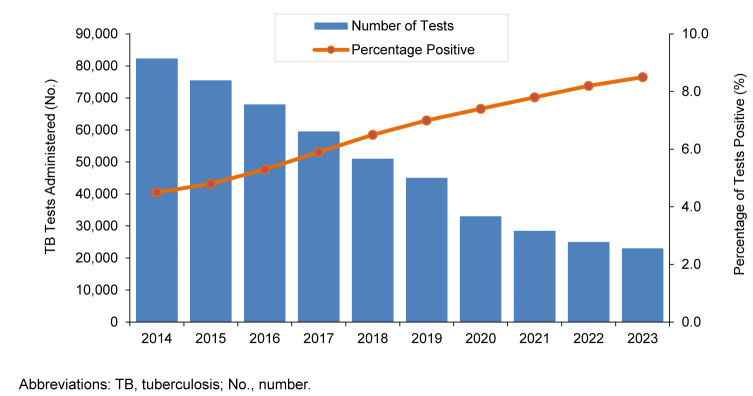
Total Number of Tuberculosis Tests Administered and Percentage of Positive Tests by Year, U.S. Army Active Component, 2014-2023

During the 10-year surveillance period (2014–2023), a total of 339,465 TB tests were administered to U.S. Army active component soldiers. Of these, 22,762 were positive, for an overall positivity proportion of 6.7%. This resulted in 18,018 individuals receiving a diagnosis of LTBI. As shown in **Figure 1** , the annual number of tests administered declined sharply over the surveillance period, from 82,295 in 2014 to 22,986 in 2023. Concurrently, the proportion of tests returning a positive result nearly doubled, showing a steady increase from 4.5% in 2014 to 8.5% in 2023.

The majority of tests were administered to soldiers who were male (n=270,057, 79.6%), non-Hispanic White (n=167,887, 49.5%), of enlisted rank (n=268,723, 79.2%), and ages 20-34 years (n=235,235, 69.3%). When evaluated by age, soldiers in the under age 20 years category had the highest positivity (8.3%); this age range represents the primary age for accession into the Army. Positivity was 7.3% for both the ages 20-24 and 30-34 years categories, followed by 6.8% for the age 25-29 years category **(Table 2)** .

**TABLE 2.** Numbers and Percentages of Tests Positive for Latent Tuberculosis and Latent Tuberculosis Cases, U.S. Army Active Component, 2014–2023

Demographic Characteristics	Total Tests	Positive Tests		LTBI Diagnoses	
	No.	No.	%	No.	%
Overall	339,465	22,762	6.7	18,018	5.3
Sex					
Male	270,057	18,005	6.7	14,254	5.3
Female	69,408	4,757	6.9	3,764	5.4
Age, *y*					
<20	42,293	3,491	8.3	2,754	6.5
20–24	97,470	7,107	7.3	5,712	5.9
25–29	81,536	5,519	6.8	4,340	5.3
30–34	56,229	4,098	7.3	3,133	5.6
35–39	31,957	1,386	4.3	1,110	3.5
40–49	26,069	1,054	4.0	877	3.4
50+	3,911	107	2.7	92	2.4
Race and ethnicity					
White, non-Hispanic	167,887	5,879	3.5	4,791	2.9
Black, non-Hispanic	63,890	7,140	11.2	5,488	8.6
Hispanic	54,957	3,796	6.9	3,070	5.6
Asian / Pacific Islander	34,511	4,494	13.0	3,528	10.2
Other	15,039	1,311	8.7	1,026	6.8
Unknown, missing	3,181	142	4.5	115	3.6
Recruit status					
Yes	80,156	11,249	14.0	8,785	11.0
No	259,309	11,513	4.4	9,233	3.6
Rank					
Enlisted	268,723	20,949	7.8	16,525	6.1
Officer	70,742	1,813	2.6	1,493	2.1
Test type					
TST	274,473	17,695	6.4	13,533	4.9
IGRA	64,992	5,067	7.8	4,485	6.9
10 leading installations
Fort Jackson, SC ^a^	38,715	5,956	15.4	4,468	11.5
USAG Yongsan-Casey, South Korea	17,191	4,533	26.4	4,043	23.5
Fort Sill, OK ^a^	23,345	3,775	16.2	3,028	13.0
Fort Leonard Wood, MO ^a^	13,821	1,239	9.0	985	7.1
Fort Benning, GA ^a^	21,638	1,228	5.7	908	4.2
USAG Bavaria, Germany	2,462	656	26.6	475	19.3
JB San Antonio, TX	15,217	463	3.0	439	2.9
USAG Hawaii	15,166	306	2.0	232	1.5
Fort Bliss, NM, TX	12,999	299	2.3	209	1.6
NSA Bethesda, MD	9,700	285	2.9	203	2.1

Abbreviations: LTBI, latent tuberculosis infection; No., number; y, years; TST, tuberculin skin test; IGRA, interferon gamma release assay; SC, South Carolina; USAG, U.S. Army Garrison; OK, Oklahoma; MO, Missouri; GA, Georgia; JB, Joint Base; TX, Texas; NM, New Mexico; NSA, Naval Support Activity; MD, Maryland.

a Installation with initial entry recruit population.

TST was the most frequently used method (n=274,473), accounting for 81.0% of all tests, while IGRAs (n=64,992) comprised the remaining 19.0% **(Table 2)** .

While men accounted for a larger absolute number of positive tests and LTBI diagnoses, the positivity proportion was nearly identical between men (6.7%) and women (6.9%) **(Table 2)** . Proportions of positive tests and LTBI diagnoses varied notably by racial and ethnic group. Asian / Pacific Islander soldiers had the highest proportions of positive tests (13.0%) and LTBI diagnoses (10.2%), followed by non-Hispanic Black soldiers (11.2% and 8.6%, respectively). In contrast, non-Hispanic White soldiers had the lowest proportions (3.5% and 2.9%, respectively) **(Table 2)** .

A noticeable difference was observed based on recruit status. The proportion of positive tests among recruits was 14.0%, compared to 4.4% among non-recruits **(Table 2)** . The IGRA test showed a slightly higher positivity proportion (7.8%) compared to the TST (6.4%). Enlisted personnel had a higher proportion of positive tests (7.8%) and LTBI diagnoses (6.1%) compared to officers (2.6% and 2.1%, respectively) **(Table 2)** .

Considerable variability in test positivity was observed between military installations **(Table 2)** . Among the 10 installations with highest LTBI test positivity, U.S. Army Garrison (USAG) Bavaria, Germany, which is the largest U.S. Army training area in Europe, comprising Grafenwoehr Tower Barracks and Hohenfels Joint Multinational Readiness Center, reported the highest proportion of positive tests (26.6%) along with USAG Yongsan-Casey in South Korea, with second-highest test positivity (26.4%). Installations that serve as large initial entry training sites, such as Fort Sill, Oklahoma (16.2%) and Fort Jackson, South Carolina (15.4%), also reported high positivity proportions. Conversely, the 10 installations with the lowest positivity for LTBI—Aviano Air Base, Italy; Barksdale Air Force Base (AFB), Louisiana; Dover AFB, Delaware; Ellsworth AFB, South Dakota; Hanscom AFB, Massachusetts; Joint Base Charleston, South Carolina; Keesler AFB, Mississippi; Kirtland AFB, New Mexico; Maxwell AFB, Alabama; and U.S. European Command—each had 0% test positivity (data not shown). This could potentially be due to effective control measures, low local TB prevalence, or even a small sample size.

## Discussion

This analysis of over 339,000 TB tests in the U.S. Army active component from 2014 through 2023 shows clear epidemiological outcomes following the 2013 MEDCOM policy ^4^ shift to targeted, risk-based screening. These findings should be viewed within the context of the greater U.S., where a diagnosis of active TB disease is relatively uncommon, with a civilian incidence rate (IR) of 2.9 cases per 100,000 persons in 2023. ^6 , 7^ This contrasts sharply with the U.S. military, where the risk is substantially lower, with an active TB disease IR estimated at less than 1 case per 100,000 persons. ^4 , 8^ Similarly, while a significant reservoir of infection exists in the U.S. general population, with an estimated 4.0% prevalence of LTBI, ^6 , 7^ the prevalence among military-age groups is estimated to be only around 1%. ^4 , 8^ The primary finding of this analysis is a sharp 72% reduction in the annual number of tests administered. The substantially higher average proportion of positive tests from 2014 to 2023 (6.7%) compared to the average from the pre-policy era of universal screening (1.3%) demonstrates the successful concentration of testing resources on those most at risk, thereby improving diagnostic yield within a low-prevalence military force.

Following the 2013 MEDCOM policy change, the decline in testing volume and corresponding rise in the positivity proportion are the expected and intended results of a successful targeted testing program. By focusing screenings on high-risk populations, such as recruits, personnel deploying to endemic areas, and close personal contacts, the policy effectively eliminated the testing of a large, low-risk population that previously diluted the overall positivity prevalence. The result is not necessarily an increase in overall LTBI within the Army, but rather an improved diagnostic yield and more efficient allocation of public health resources, a finding consistent with the stated goals of the policy.

The demographic and military characteristics associated with LTBI in this analysis are largely consistent with previous reports, ^3 , 5^ although the magnitude of these associations is more pronounced due to targeted testing. The elevated proportion of positive tests among recruits (14.0%) underscores that accession screening remains critical for identifying prevalent LTBI acquired prior to service. The disparities observed among racial and ethnic groups, particularly the high proportions among non-Hispanic Black (11.2%) and Asian / Pacific Islander (13.0%) soldiers, are also consistent with national trends. ^6 , 9^

These associations are likely confounded, however, by socio-economic factors and, most importantly, country of origin. Non-U.S. birth is a primary LTBI risk factor, and it is probable that this unmeasured variable accounts for a significant portion of the observed differences between racial, ethnic, and even rank categories. ^6 , 10^ The higher proportion of LTBI among enlisted personnel compared to officers, for example, is more likely a reflection of underlying demographic differences at accession than of occupational exposures during service.

The pronounced disparities among racial and ethnic groups warrant further consideration, particularly considering this analysis's limitations. The absence of data on country of birth is a significant confounding variable that likely explains a substantial portion of observed differences. National data consistently show that a majority of TB cases in the U.S. occur in non-U.S. born individuals. ^9^ It is highly plausible that the elevated LTBI proportions among Asian/Pacific Islander and non-Hispanic Black soldiers are more reflective of a higher prevalence of non-U.S. birth within those cohorts than of any inherent racial or ethnic predisposition. Future surveillance should aim to integrate country of birth data into the initial screening process, which would enable more precise risk stratification, distinguishing risk acquired prior to service from that acquired during a military career. New country of birth data would allow public health officials to design prevention and treatment strategies more effectively.

From a policy perspective, while the targeted screening strategy has proven successful in enhancing diagnostic yield, these findings highlight the ongoing need for vigilance. The high prevalence of LTBI identified in recruits (14.0%) confirms that the point of accession is the most critical juncture for TB control within the Army. Furthermore, the slight but notable difference in positivity between IGRA (7.8%) and TST (6.4%) tests suggests opportunity for policy refinement; this variance could be attributable to IGRA's greater specificity, especially among individuals who may have received the Bacille Calmette-Guérin vaccine, or it may reflect its use in more selectively high-risk groups. Given these factors, the Army may consider recommending IGRA as the primary screening tool for specific high-risk recruit populations, such as those born in TB-endemic countries, to further optimize the accuracy and effectiveness of the TB control program.

There are several limitations to this analysis. First, the demographics of the 2 periods (all forces vs. Army), living conditions, and potential exposures in different geographic locations may contribute to some differences. Second, there are generalizability limitations, as results are specific to the U.S. Army active component, limiting the relevance to other MHS beneficiaries such as other service components, family dependents, and retirees. Third, the dataset lacked information on service members' countries of birth, a crucial unmeasured confounder that, as discussed, likely influenced observed associations with race and ethnicity. Fourth, there are data completeness problems, as the race and ethnicity data had 6% unknown or missing entries, potentially biasing disparity analyses. Fifth, the definition of an LTBI case relied on the presence of an ICD-9-CM / ICD-10-CM code within 30 days of a positive test. This is a significant assumption, as administrative or clinical lapses may lead to misclassification; it is possible that some individuals with a positive test did not receive a corresponding diagnostic code, or vice versa, potentially leading to an under-estimation of the true LTBI burden. Sixth, this analysis assumes uniform implementation of the 2013 MEDCOM policy, but adherence likely varied over time and between installations. This inconsistent application of targeted testing could contribute to the variability in positivity and may have influenced the overall trends. Finally, these are observational data, so causality cannot be determined; external factors, such as changes in deployment patterns or recruitment demographics, may also have influenced the observed trends.
